# iTRAQ-Based and Label-Free Proteomics Approaches for Studies of Human Adenovirus Infections

**DOI:** 10.1155/2013/581862

**Published:** 2013-03-11

**Authors:** Hung V. Trinh, Jonas Grossmann, Peter Gehrig, Bernd Roschitzki, Ralph Schlapbach, Urs F. Greber, Silvio Hemmi

**Affiliations:** ^1^Institute of Molecular Life Sciences, University of Zurich, Winterthurerstrasse 190, 8057 Zurich, Switzerland; ^2^Life Science Zurich Graduate School, Molecular Life Science Program, Switzerland; ^3^Functional Genomics Center Zurich, University of Zurich, Winterthurerstrasse 190, 8057 Zurich, Switzerland

## Abstract

Both isobaric tags for relative and absolute quantitation (iTRAQ) and label-free methods are widely used for quantitative proteomics. Here, we provide a detailed evaluation of these proteomics approaches based on large datasets from biological samples. iTRAQ-label-based and label-free quantitations were compared using protein lysate samples from noninfected human lung epithelial A549 cells and from cells infected for 24 h with human adenovirus type 3 or type 5. Either iTRAQ-label-based or label-free methods were used, and the resulting samples were analyzed by liquid chromatography (LC) and tandem mass spectrometry (MS/MS). To reduce a possible bias from quantitation software, we applied several software packages for each procedure. ProteinPilot and Scaffold Q+ software were used for iTRAQ-labeled samples, while Progenesis LC-MS and ProgenesisF-T2PQ/T3PQ were employed for label-free analyses. *R* ^2^ correlation coefficients correlated well between two software packages applied to the same datasets with values between 0.48 and 0.78 for iTRAQ-label-based quantitations and 0.5 and 0.86 for label-free quantitations. Analyses of label-free samples showed higher levels of protein up- or downregulation in comparison to iTRAQ-labeled samples. The concentration differences were further evaluated by Western blotting for four downregulated proteins. These data suggested that the label-free method was more accurate than the iTRAQ method.

## 1. Introduction

Quantitative proteomics based on mass spectrometry (MS) is an important methodology for biological and clinical research allowing, for example, the identification of functional modules and pathways, or the monitoring of disease biomarkers [1, 2]. Relative quantitation of two or more samples for studies of differential protein expression is of particular importance. Quantitative results can be gained using stable isotope labels or label-free methods [3–5]. In general, isotope labels offer higher reproducibility in quantitation, and label-free methods require highly reproducible LC-MS/MS platforms [3]. Several labeling methods based on heavy isotopes such as ^2^H, ^13^C, ^15^N, and ^18^O have been developed and allow relative quantitation using MS. *In vivo* metabolic labeling methods such as stable isotope labeling by amino acids in cell culture (SILAC) were introduced for arginine [6], lysine [7], tyrosine [8], or leucine [9]. For direct labeling of proteins or peptides, two strategies are being generally used. Isotope coded affinity tag (ICAT) labeling allows enrichment and MS analysis of cysteine-containing peptides [10]. iTRAQ was developed for both relative and absolute quantitation using internal peptide standards [11]. The iTRAQ reagents react with primary amines of amino-termini or lysine residues and hence label most peptides and proteins occurring in cells. Upon collision-induced dissociation (CID), iTRAQ reporter ions are released and their relative intensities are used for protein quantitation. In contrast to ICAT and SILAC, where two or three samples are compared, iTRAQ allows simultaneous labeling and quantitation of four or eight samples [11, 12]. By combining multiple samples in one run, the instrument time for analyses can be reduced, and variations between different LC/MS runs do not affect the results. Comparative studies for different isotope labels including differential gel electrophoresis (DIGE), ICAT, and iTRAQ showed that iTRAQ is more sensitive than ICAT [13]. Another study compared iTRAQ-label and label-free methods and identified 79 proteins with both methods [14], but it remains unclear which method is best suited for quantitative proteomics. However, a recent analysis of two *Chlamydomonas reinhardtii* strains by Wang et al. provided a substantial comparison between iTRAQ-based and label-free methods [15]. The results indicated that both methods were comparable although quantitation for spiked-in standards reached closer to the expected values in label-free quantitation experiments, and most significantly regulated proteins showed slightly higher changes by label-free quantitation compared to iTRAQ-label based quantitation.

High-throughput quantitative proteomics experiments produce large datasets. To quantify iTRAQ ratios, an array of bioinformatic tools was introduced, including ProQuant (Applied Biosystems), TandTRAQ [16], Multi-Q [17], Mascot 2.2 (Matrix Science, London, UK), Scaffold Q+ (Sc+, http://www.proteomesoftware.com/), and ProteinPilot (PP) [18]. PP utilizes Paragon as a search algorithm. Unlike PP, Scaffold does not contain a search engine but uses Bayesian statistics and search outputs, such as Mascot to estimate peptide and protein identification probabilities. Scaffold has recently been updated to the Sc+ version with enhanced features for iTRAQ quantitation. Although iTRAQ-labeling has been widely applied, there is an ongoing discussion about the accuracy of the deduced protein quantitations, particularly when sample mixtures are highly complex [19–21]. iTRAQ-labels typically reveal fold changes of less than 2 orders of magnitude [22], unlike microarrays, which can be utilized for expression profiling over 3 orders of magnitude. This may be perceived as a limitation of the iTRAQ-labeling method for quantitative proteomics.

Label-free approaches can be applied for both shotgun and targeted proteomics [23]. Moreover, they are cost effective and reproducible [24]. There are two general approaches for label-free quantitation, measurement of spectral peak intensities [25] and spectral counting [26]. Both approaches require extensive processing of raw LC/MS data, leading to high demands on the bioinformatic tools. Thus, multiple software packages are recommended for data analyses. For instance, Progenesis LC-MS (PL, Nonlinear Dynamics) uses vectors to match all experiments to one reference sample for easy comparison of results. Next, a global scaling factor for each LC-MS run is estimated to normalize all runs. The peptide abundance is taken as the sum of the peak areas within the isotope boundaries while the protein abundance is the sum of the abundance of all peptides from one particular protein. Finally, the peak lists are exported in the mgf format and can be used for the Mascot search engine and are later imported back into PL. In addition, the counting of spectrum-peptide matches is often not an accurate measure of protein abundance due to physicochemical properties of peptides and the local chemical environment [27, 28]. To overcome a bias of MS/MS spectral counting, Lu et al. developed a so-called “Absolute Proteomics Expression” counting method by introducing correction factors to predict detection rates of peptides [29]. More recently, Grossmann et al. refined a procedure for label-free quantitation by selecting the top N most prevalent precursor ions per protein (TNPQ), where N is equal to 2 or larger [30]. 

In this study, we compared the iTRAQ-labeling method with the label-free method in complex samples from human lung adenocarcinoma cells infected with human adenovirus type 3 (HAdV-B3) or type 5 (HAdV-C5). HAdVs are significant pathogens causing respiratory disease, gastroenteritis, acute hemorrhagic cystitis, meningoencephalitis, or conjunctivitis [31, 32]. HAdVs are widely used and the most extensively studied viruses for gene delivery/therapy applications [33–36]. HAdV-C5 belongs to the best-studied viruses. It binds to the coxsackie and adenovirus receptor (CAR) [37, 38], internalizes by receptor-mediated endocytosis, and activates its uncoating program at the cell surface [39, 40]. For infection of polarized cells, these viruses use cytokine-controlled receptor expression on the apical surface [41] and deliver their DNA genome by uncoating at the nuclear pore complex [42]. HAdV-B3 is a representative of the species B types that utilize CD46 and/or desmoglein 2 (DSG2) as attachment receptors [39, 43, 44]. The underlying complexity of host factors controlling virus entry and infection is, however, incompletely understood [45]. Accurate analyses of global protein expression patterns in infected cells can contribute to a better understanding of essential virus-host interactions. 

## 2. Methods

### 2.1. Cells, Virus, and Cell Lysate Preparation

Human A549 lung adenocarcinoma epithelial cells were cultured in Dulbecco's modified Eagles medium (Sigma) supplemented with 8% fetal bovine sera. A549 cells were infected with HAdV-B3 or HAdV-C5 using a multiplicity of infection (MOI) of 200 infectious virus particles/cell. Noninfected cells (ctrl) and HAdV-B3/C5-infected cells were harvested at 24 hours (h) postinfection (p.i.) with biological duplicates for each condition. The cells were washed twice in PBS by centrifugation. The cell pellets were then resuspended in lysis buffer containing 10 mM Hepes pH 7.4, 150 mM NaCl, 1% NP-40, 0.5% sodium deoxycholate, 0.1% SDS, and protease inhibitor cocktail (Roche) with sonication. Nonsolubilized material was removed by centrifugation at 16,000 ×g for 20 minutes (min). The proteins contained in the supernatant were precipitated by the addition of TCA to 20% and twice washed with 100% acetone. Finally, the proteins were solubilized in 0.5 M triethylammonium bicarbonate pH 8.5 plus 0.2% SDS, 1 M urea, and 15% methanol. Protein concentrations were measured by the Qubit method (Invitrogen). For downstream analysis by LC-MS/MS, 30 *μ*g of individual protein samples was used. 

### 2.2. Protein Digestion, iTRAQ 8plex Labeling, and Chromatography

For these experiments, 30 *μ*g proteins of each sample was reduced in 2 mM of TCEP at 37°C for 1 h, and the cysteine residues were blocked in 10 mM MMTS at room temperature for 15 min followed by trypsin digestion (modified trypsin, Promega) at a protease : protein ratio of 1 : 12.5 (w/w) at 37°C for 8–10 h. iTRAQ-8plex labeling reagents (Applied Biosystems) were added to the peptide samples, which were incubated at room temperature for 140 min. The reaction was stopped by the addition of 10 mM KH_2_PO_4_, 25% ACN, pH 2.6 (solvent A), followed by centrifugation at 16,000 ×g for 10 min to remove the aggregated proteins. The digested protein samples were separated by using multidimensional liquid chromatography. In the first dimension, the peptide mixtures were fractionated using an SCX column (Polysulphoethyl A, 2.1 mm inner diameter, 200 mm length, 300 Å pore size, 5 *μ*m particle size, PolyLC Inc.). A linear binary gradient from solvent A (10 mM KH_2_PO_4_, 25% ACN, pH 2.6) to solvent B (10 mM KH_2_PO_4_, 0.35 M KCl, 25% ACN, pH 2.6) was applied: 0% to 5% solvent B in 15 min, 5% to 35% B in 35 min, and 35% to 100% B in 10 min. The entire run lasted 90 min, and 27 SCX fractions were collected. These fractions were vacuum-dried and redissolved in 0.1% TFA, 5% ACN. Based on the SCX chromatograms, the 27 SCX fractions were combined into 8 pools. All pools were further desalted by Sep-Pak C_18_ columns (Waters). 

Next, the pooled SCX fractions were automatically injected by a Famos autosampler and separated by an UltiMate capillary LC system (Dionex/LC Packings) and loaded onto a C_18_ PepMap main column (75 *μ*m ID, 150 mm length, 100 Å pore size, and3 *μ*m particle size; Dionex) using a linear binary gradient (solvent A: 0.1% TFA; solvent B: 0.1% TFA, 80% ACN). HPLC linear gradients were increased by solvent B from 0% (10 min) to 50% (100 min) and from 50% to 100% (112 min). The peptides eluting from the LC column were then mixed with 3-4 mg/mL of CHCA matrix (Bruker Daltonics) in 0.1% TFA, 70% ACN containing internal neurotensin peptide (Sigma) and were automatically deposited onto an Opti-TOF LC MALDI plate (Applied Biosystems) by using a Probot spotting device. Mass spectrometric analysis was conducted with a 4800 MALDI-TOF/TOF instrument (Applied Biosystems).

### 2.3. Sample Preparation and Chromatography for Label-Free Experiments

For the label-free approach, 30 *μ*g of proteins for each replicate were reduced with 5 mM of TCEP at 37°C for 1 h and blocked with 10 mM iodoacetamide at room temperature for 30 min followed by trypsin digestion at 37°C for 8–10 h. The trypsin digestion was stopped by adding 5% TFA, and the pH value was adjusted by 10 mM KH_2_PO_4_, 25% ACN, pH 2.6. The aggregated proteins were removed by centrifugation at 16,000 ×g for 10 min. The protein digests were purified by using Sep-Pak C_18_ columns (Waters). The desalted peptides were vacuum-dried and dissolved in 0.2% formic acid and 3% ACN. The samples were injected into an Eksigent-nano-HPLC system (Eksigent Technologies, Dublin CA, USA) by an autosampler and separated on a self-made RP tip column (75 *μ*m × 80 mm) packed with C_18_ material (3 *μ*m, 200 Å, AQ, Bischoff GmbH, Leonberg, Germany). The column was equilibrated with 97% solvent A (A: 1% ACN; 0.2% formic acid in water) and 3% solvent B (B: 80% ACN, 0.2% formic acid in water). Peptides were eluted using the following gradient: 0–50 min, 3–30% B; 50–58 min, 30–50% B and 58–60 min, 50–97% B at a flow rate of 0.2 *μ*L/min.

### 2.4. Mass Spectrometry Analysis

Mass spectrometric analysis of the iTRAQ-labeled samples was performed on a 4800 MALDI-TOF/TOF instrument equipped with a 355 nm Nd:YAG laser. Mass spectra were acquired in positive reflectron mode in the mass range from m/z 850 to 4,000, with a focus mass of m/z 2,100. They were generated by accumulating data from 600 laser pulses, and they were internally recalibrated based on the molecular mass of the neurotensin peptide. The ten most intensive peptide ion signals showing an S/N ratio > 100 were subjected to MS/MS acquisition. Peptide CID was conducted at collision energy of 1 kV and at a gas pressure of approximately 2.5 × 10^−6^ Torr. 

For the label-free approach, high accuracy mass spectra were acquired on an LTQ-ICR-FT-Ultra mass spectrometer (Thermo Scientific, Bremen, Germany) in the mass range of 300–2,000 m/z and at a target value of 1 × 10^6^ ions and a resolution of 100,000 at m/z 400. Up to five data dependent MS/MS were recorded in parallel in the linear ion trap from the most intense ions with charge states 2+ or 3+ using collision induced dissociation. Target ions already selected for MS/MS acquisition were dynamically excluded for 120 seconds. Three independent LC/MS runs were performed for each sample.

### 2.5. Database Searches and Quantitative Proteome Analysis

Both PP and Mascot search engines were used for protein identification from iTRAQ data acquired on the MALDI-TOF/TOF instrument. Only Mascot was utilized to identify proteins from data acquired on the LTQ-FT-ICR instrument for label-free quantitation. For the analysis with PP v3.0 (Applied Biosystems), the acquired data was directly fetched from the Oracle database and searched with the Paragon algorithm. For the analyses with Mascot, peak lists (mgf files) were generated using Mascot Distiller software v2.3 (Matrix Science Ltd., London, UK). The same database, which contains a nonredundant protein database for both human and HAdVs proteins (http://www.uniprot.org/) and a few of HAdV entries derived from NCBI, was applied for both PP and Mascot searches. The combined database contains 41,135 entries in total including the concatenated reversed decoy entries, which were added in order to estimate the protein FDR. The FDR was calculated according to [46]. 

In Mascot searches, tolerances of 25 ppm for peptide masses and 0.25 Da for fragment ions were specified for data obtained by using the MALDI-TOF/TOF instrument. In case of data obtained by LTQ-FT-ICR analyses, tolerances of 5 ppm for peptides and of 0.6 Da for fragment ions were used. In all searches, carbamidomethylation or MMTS modification of cysteine residues was selected as fixed modifications, and oxidation of methionine was considered as variable modification. To obtain a protein FDR below 5%, a Mascot ion score ≥30 for peptide identifications was required. 

### 2.6. Software Used for Protein Quantitation

PP and Sc+ (Proteome Software) were used for protein quantitation of iTRAQ-labeled samples. For PP, bias and background correction was applied, and biological modifications were allowed. To obtain a protein FDR below 5%, protein identifications were filtered with PP score ≥1.3 (equivalent to 95% confidence). PP analysis provided both protein identification and quantitation results. By contrast, we only used the protein identification feature from Mascot searches and applied the quantitation feature from Sc+. To improve reliability and confidence of protein quantitation, all precursor ion signals showing an intensity ≤ 50 were discarded before the data were imported into Sc+. We used two rather different software products for the comparison of the iTRAQ data also to investigate the effect of lower scoring peptide assignments on protein quantitation. PP includes peptides with relatively low scores in protein quantitation, while Sc+ is very stringent and takes only confident identifications into account for quantification of proteins.

The quantitative analyses for the label-free approaches were performed by using the commercial PL software (Nonlinear Dynamics) as recommended by the vendor, or the Progenesis feature data export was combined with the emerging “high flyer” strategy to quantify proteins based on LC-MS signals. The idea of this strategy termed ProgenesisF-T2PQ (PF2) or ProgenesisF-T3PQ (PF3) [30] is that irrespective of how many peptides are found for one particular protein; only the most intense (*n*) precursor signals are used for protein quantitation. We adapted this method referred to as PF2 or PF3, respectively, based on the aligned Progenesis feature map by averaging the top N normalized volumes of features from the same protein. For the Progenesis analysis, which served also as the base for the ProgenesisF-T2(3)PQ, the raw files were imported and the automatic choice for the reference run for the aligning was accepted. For each file, we seeded manually three to five vectors before automatic aligning to give the algorithm a good starting point. The aligned LCMS map was filtered with respect to the retention time, and only the relevant part of the gradient was retained for quantitation. The identification results were filtered with the same ion score ≥30. Conflicting features were not used in quantitation. 

To calculate the log_2_ ratio for proteins, in the first stage, we ranked the features of one protein according to their mean abundance from the two biological replicates. Then, the top 2 or top 3 features were averaged for each condition (HAdV-B3/C5-infected and noninfected) to get the quantitative value for each protein for each condition. Next, we calculated the log_2_ ratio for each protein. Proteins with log_2_ ratios either ≥0.6 or ≤ −0.6 were considered to be differentially expressed. No Student's*t*-test or statistical analysis was applied.

### 2.7. Western Blot Analysis

40–60 *μ*g/lane of biological replicate A549 cell lysates including noninfected and HAdV-B3/C5-infected cells was separated on 10–15% SDS-PAGE and blotted onto Immobilon PVDF transfer membranes (Millipore) using the semidry transfer system (Biorad). The rabbit antihuman Gal1 and Gal3 antibody were generous gifts from Professor Walter Nickel (University of Heidelberg, Germany). The mouse monoclonal antibody with specificity for anterior gradient protein 2 (AG-2, cat: sc-101211) and the goat polyclonal antibody with specificity for 4F2 cell-surface antigen heavy chain (CD98, cat: sc-31251) were purchased from Santa Cruz Biotechnology Inc. The mouse anti-*α*-tubulin monoclonal antibody (DM1A) was purchased from Sigma. All primary antibodies were diluted 1 : 1,000, except for DM1A, which was diluted 1 : 5,000. Unconjugated rabbit anti-goat IgG was diluted 1 : 3,000 (cat: G 4018, Sigma), and secondary HRP conjugated anti-mouse antibody (cat: NA931V, GE Healthcare) and anti-rabbit antibody (cat: NA934V, GE Healthcare) were used at a 1 : 3,000 dilution. Signal detection was performed in the chemiluminescence scanning mode of Image reader LAS 3000 (FUJIFILM Science Lab), and signal quantitation was performed using Image Gauge version 3 (FUJIFILM Science Lab). 

## 3. Results

### 3.1. Workflow for Quantitative Proteomics Using iTRAQ-Based and Label-Free Methods

**Figure 1:**
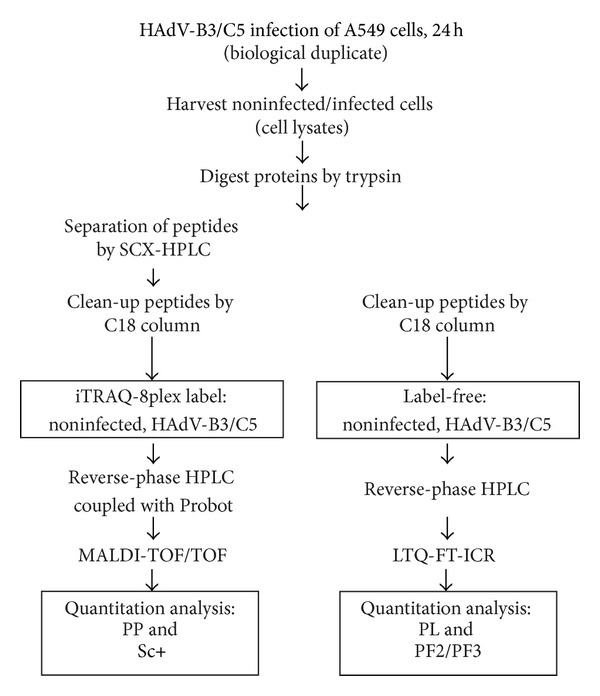
Workflow for comparisons of iTRAQ-label and label-free quantitations. A549 cell lysates were harvested 24 h post infection (p.i.), and proteins were precipitated by TCA, followed by trypsin digestion. For the iTRAQ-label approach, the tryptic peptides were labeled with iTRAQ-8plex reagents. These included biological replicates for noninfected cells (reporter 113 and 114), for HAdV-B3-infected cells (reporter 115 and 116), for HAdV-C5-infected cells (reporter 117 and 118), and for HAdV-B35-infected cells (reporter 119 and 121, not shown here). Peptides were separated by SCX chromatography following RP chromatography and spotted onto MALDI plates. The mass spectrometric analyses were performed on the MALDI-TOF/TOF instrument. Protein quantitation based on iTRAQ data was performed with PP and Sc+ software. For the label-free approach, protein digests were analyzed with LC-MS/MS without prefractionation. The obtained data was quantified by using PL and PF2 and PF3.

Two independent workflows for iTRAQ and label-free analyses were used with lysates of noninfected and HAdV-B3- or HAdV-C5-infected A549 cells (Figure 1). For the iTRAQ-labeled samples, we used PP and Sc+ software, and for the label-free samples, PL and ProgenesisF-T2PQ (PF2) and ProgenesisF-T3PQ (PF3) software. For PP, 1,538 proteins were commonly quantified for HAdV-B3- and HAdV-C5-infected cells compared with noninfected cells (Table 1), and a false discovery rate (FDR) for protein identification of 1.89% was indicated. For Sc+, the numbers of quantified proteins were below those from PP analyses with 1,340 commonly quantified proteins. For the Sc+ output, the FDR was zero. Together, these results indicated high levels of protein identification reliability. 

### 3.2. Correlation Analyses for iTRAQ Quantitation Using PP and Sc+ Software

**Figure 2:**
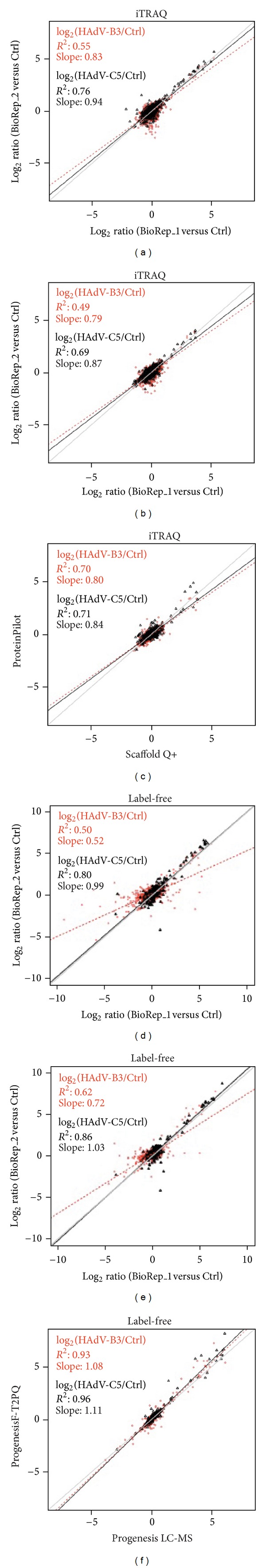
Reproducibility of two independent biological replicates using either iTRAQ-label or label-free method and comparison within the iTRAQ-label or label-free quantitation with different software. Different expression of proteins in HAdV-B3-infected cells (red) and HAdV-C5-infected cells (black) is shown based on log_2_ ratio. For the iTRAQ-label approach, Scatter plots for quantified proteins were analyzed by PP (a) and Sc+ (b), and comparison of software included PP versus Sc+ (c). For the label-free approach, Scatter plots for quantified proteins were analyzed by PL (d) and by PF2 (e), and comparison of software included PL versus PF2 (f). For comparisons in (c) and (f), both biological replicates were used.

We performed ratiometric analyses of protein abundance between infected and noninfected cells to estimate the steady state of the cellular and viral proteome 24 h post infection when most of the viral proteins are expressed and the infection progresses towards the lytic phase [47]. First, we examined the reproducibility of two biological replicates from iTRAQ analyses quantified by PP and Sc+. By PP analyses, the squared Pearson correlation analysis revealed an *R* ^2^ of 0.55 for HAdV-B3-infected cells, and an *R* ^2^ of 0.76 for HAdV-C5-infected cells, respectively (Figure 2(a)). By Sc+ analyses, the *R* ^2^ coefficient was 0.49 for HAdV-B3-infected cells and 0.69 for HAdV-C5-infected cells, respectively (Figure 2(b)). The *R* ^2^ Pearson correlations between PP and Sc+ were 0.7 for HAdV-B3, and 0.71 for HAdV-C5-infected cells (Figure 2(c)). This indicated good reliability of both software tools, independent from the algorithm or search engine used for quantitation. These data also showed that the expression changes were more pronounced by Sc+ than PP analyses, as indicated by the slope values, which are smaller than 1 in the Sc+ versus PP plots for HAdV-B3 and HAdV-C5 (Figure 2(c)), red and black, resp.). This may be due to the fact that PP is also taking lower confident peptides into account for quantitation, while for Sc+ only high confidence peptide assignments are used for quantitation. 

Importantly, most of the proteins quantified by Sc+ were also found by using PP (Table 2, see Supplementary Table 1 in supplementary material available online on http://dx.doi.org/10.1155/2013/581862 pages 82–119). We quantified 1,267 and 1,263 proteins in common for both PP and Sc+ in HAdV-B3 and HAdV-C5-infected cells, respectively. The ratios of most quantified proteins were similar for PP and Sc+. For 65.5% of the proteins (827 out of 1,263) from HAdV-B3-infected cells, the ratios between PP and Sc+ had variations of about 20%, and for HAdV-C5-infected cells, 64.6% (819 out of 1,267 proteins) had 20% variations. 

It is of note that viral proteins were exclusively detected in infected but not in noninfected cells. The upregulated proteins consisted of viral and cellular proteins in both PP and Sc+ analyses. When using PP, 37 proteins were found up-regulated with a threshold of 0.6 (log_2_ ratio equivalent to 1.5-fold changes) in HAdV-B3-infected cells, and 51 proteins were downregulated in these cells (S Table 2). Likewise, for HAdV-C5-infected cells, 33 proteins were up-regulated, and 20 proteins were downregulated. Within Sc+, 31 proteins were found up-regulated and 50 proteins were downregulated in HAdV-B3-infected cells, while 23 proteins were up-regulated and 25 proteins were downregulated in HAdV-C5-infected cells. Altogether, 74 proteins were found up- or downregulated with both programs in HAdV-B3-infected cells, and 40 proteins changed by more than 1.5-fold by both analysis programs for HAdV-C5-infected cells. Twelve and five proteins were single-hit detections with PP or Sc+ in HAdV-B3-infected cells, and eighteen and nine viral proteins in HAdV-C5-infected cells. These differences are likely due to a different scoring algorithms used by the respective software programs. 

Ambiguous results between PP and Sc+ analyses were obtained for one of 1,263 quantified proteins in HAdV-B3-infected cells, and two of 1,267 proteins in HAdV-C5-infected cells (S Table 2). For HAdV-B3, the ambiguously classified protein was eukaryotic translation initiation factor 3 subunit F (EIF3F, O00303), which had a log_2_ ratio of −1.12 with PP and −0.6 with Sc+. These differences were likely due to the filtering parameters of PP and Sc+. In the Sc+, both ion precursors and their MS/MS fragmentations are used for protein quantitation while in the PP only one ion precursor and its MS/MS fragmentation are considered. For EIF3F, the two ion precursors had different values explaining the discrepancy between PP and Sc+. Similarly, two different precursors were used for PP quantitation in HAdV-C5-infected cells, and one precursor ion was taken into account for Sc+ quantitation in the case of sterol-4-alpha-carboxylate 3-dehydrogenase, (NSDHL, Q15738), which was scored as 1.1 of log_2_ ratio change in PP, and without change in Sc+. The second protein misclassified in HAdV-C5 was DNA polymerase epsilon subunit 4. PP analyses showed upregulation by 0.85 of log_2_ while Sc+ indicated downregulation by −0.90 (S Table 2). Unlike the two cases described above, both precursor ions were scored in PP and Sc+ quantitation, but two precursor ions had low intensity of reporter ions, which created a bias in the PP quantitation and hence caused the difference in software output. 

### 3.3. Label-Free Quantitation by PL, PF2, and PF3 Quantitation Software Yields Similar Results

For analyses of the label-free data, we also utilized several quantitation algorithms, including PL, and PF2 and its variant PF3. These programs commonly use Mascot as search engine for protein identification but apply different algorithms for protein quantitation. With PL quantitation, we obtained results for 661 and 660 proteins in HAdV-B3 and HAdV-C5-infected cells, respectively. The reproducibility of two biological replicates was lower in HAdV-B3-infected cells (Pearson correlation *R* ^2^ of 0.50) than in HAdV-C5-infected cells (*R* ^2^ value of 0.80) as shown in Figure 2(d) and S Table 1. Among the quantified proteins, 48 were found up-regulated (≥0.6 log_2_ ratio) in HAdV-B3-infected cells, including 16 viral proteins, and 39 proteins were downregulated (≤−0.6 log_2_ ratio) (S Table 2). For HAdV-C5-infected cells, 59 proteins were found to be up-regulated, including 17 viral proteins, and five proteins were downregulated.

The label-free data were also quantified by using our own high-flyer strategy PF2 and PF3. With PF2, 439 proteins for both HAdV-B3- and HAdV-C5-infected cells were quantified with good correlation coefficients in replica tests with *R* ^2^ of 0.62 for HAdV-B3-infected cells, and 0.86 for HAdV-C5-infected cells (Figure 2(e) and S Table 1). Proteins that were either downregulated (≤−0.6 log_2_ ratio) or up-regulated (≥0.6 log_2_ ratio) are presented in S Table 2. Using PF2, 42 proteins were found to be up-regulated in HAdV-B3-infected cells, including 16 viral proteins, and 37 proteins appeared as downregulated. In HAdV-C5-infected cells, 54 proteins were found to be up-regulated, including 17 viral proteins, and seven proteins were downregulated. 17 of the regulated proteins were found in both HAdV-B3- and HAdV-C5-infected cells. 

Using PF3, we quantified 347 proteins in both HAdV-B3- and HAdV-C5-infected cells (S Table 1). The comparison of the data obtained with PF2 and PF3 revealed a squared Pearson correlation *R* ^2^ of 0.97 for proteins from HAdV-B3-infected cells and *R* ^2^ of 0.99 for proteins of HAdV-C5-infected cells (S Figure 1 and S Table 1), indicating excellent correlation between PF2 and PF3. Since PF2 had higher sensitivity than PF3, we subsequently used PF2 for label-free analyses.

When we compared the squared Pearson correlation between PL and PF2 data, the resulting correlation *R* ^2^ amounted to 0.93 for proteins of HAdV-B3-infected cells, and 0.96 for proteins of HAdV-C5-infected cells for 439 common proteins (Figure 2(f) and S Table 1, pages 34–47). In HAdV-B3-infected cells, 78, including 16 viral proteins, were up- or downregulated (0.6 log_2_ ratio cutoff). In HAdV-C5-infected cells 57 proteins were up- or downregulated (0.6 log_2_ ratio cutoff), including 16 viral proteins. These results indicated a high reproducibility of biological replicates analyzed by either PL or PF2 software. In summary, the differential expression patterns of cellular proteins found in HAdV-B3/C5-infected cells compared to noninfected cells correlated well when analyzed by the two programs. The differences in protein abundance between HAdV-B3- and HAdV-C5-infected cells are likely due to the different nature of the viruses rather than due to different analysis software or algorithms. 

### 3.4. Correlation Analysis for iTRAQ-Based versus Label-Free Quantitation

**Figure 3:**
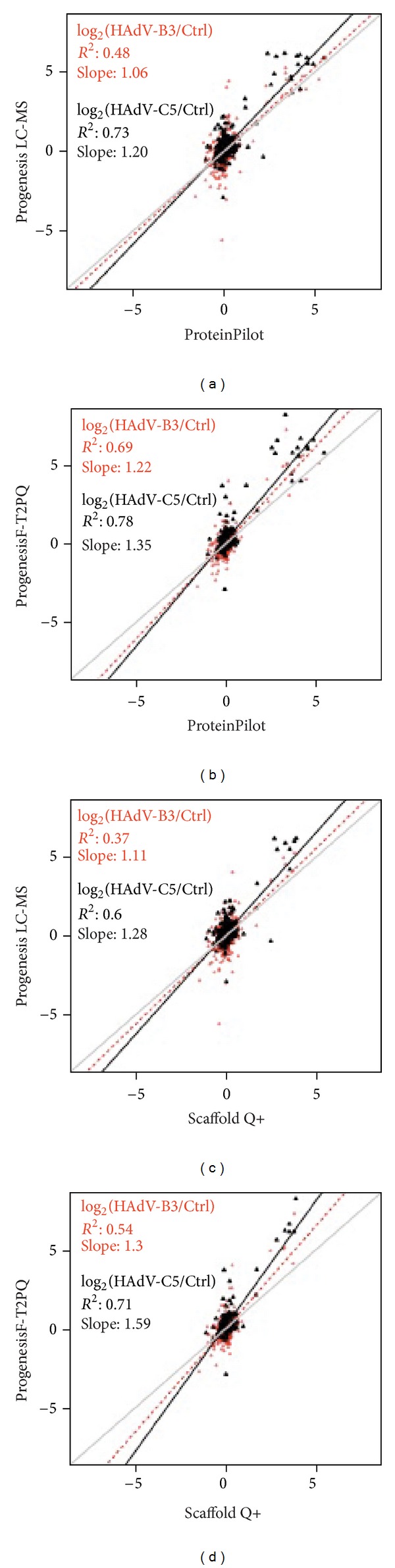
Comparisons of iTRAQ-label-based quantitation versus label-free quantitation. All four comparisons are given by log_2_ ratios with HAdV-B3-infected cells (red) and HAdV-C5-infected cells (black). Scatter plot analysis for quantified proteins was performed by PP versus PL (a), PP versus PF2 (b), Sc+ versus PL (c), and Sc+ versus PF2 (d). As in Figure 2, both biological replicates were used.

We next addressed the level of correlation between iTRAQ-label and label-free methods by comparing four data sets, PP against PL, PP against PF2, Sc+ against PL, and Sc+ against PF2 (Figures 3(a)–3(d)). For iTRAQ-labeled samples from HAdV-B3- or HAdV-C5-infected cells we found a correlation coefficient *R* ^2^ between PP and PL of 0.48 and 0.73, considering 564 and 569 commonly quantified proteins, respectively (Figure 3(a) and S Table 1, pages 1–18). Among these, 23 proteins, including 12 viral proteins, were up- or downregulated in HAdV-B3-infected cells, with both, iTRAQ-8plex (by PP) and label-free (by PL) procedures, and 18 proteins in HAdV-C5-infected cells (S Table 2). We noticed that all proteins above a cutoff of 0.6 log_2_ ratio had a similar pattern of either up- or downregulation. However, the fold change for the same proteins quantified by label-free methods generally gave higher values than the fold change quantified by quantified by the iTRAQ-label method. This observation was confirmed by slope values of 1.06 for proteins of HAdV-B3-infected cells and 1.20 for proteins of HAdV-C5-infected cells (Figure 3(a)). This is illustrated for instance with the viral proteins from these samples (S Table 2). 

Most of the cellular proteins with expression changes above the 0.6 log_2_ ratio cutoff revealed the same trend with both analysis programs, except two proteins, P62633 (cellular nucleic acid-binding protein (CNBP)) and P68036 (ubiquitin-conjugating enzyme E2 L3 (UBE2L3)). The former was downregulated according to the iTRAQ ratio (by −0.74 log_2_ ratio) but up-regulated in the label-free analysis (by 1.01 log_2_ ratio) in HAdV-B3-infected cells. The latter was up-regulated according to the iTRAQ analysis (by 0.88 log_2_ ratio) and downregulated according to the label-free quantitation (by −1.34 log_2_ ratio) in HAdV-B3-infected cells. In these cases, precursor ions resulted in low intensities, and only one precursor ion was used for quantitation. In these two cases, it is difficult to judge which quantitation method is more reliable. 

The squared Pearson correlations of PP (iTRAQ-label) and PF2 (label-free) gave rise to higher *R* ^2^ values compared to the PP and PL pair, that is 0.69 for HAdV-B3 from 406 proteins, and 0.78 for HAdV-C5 from 410 proteins (Figure 3(b) and S Table 1 page 58–70). Among these, 22 and 18 proteins were up- or downregulated (log_2_ ratio ≥ 0.6) in both iTRAQ-8plex (PP) and label-free analyses (PF2) for HAdV-B3- and HAdV-C5-infected cells, respectively (S Table 2). Similar to the findings in the comparison of PP against PL, fold changes of protein expression levels were more pronounced for the data derived by PF2 compared to PP, as illustrated by slope values of 1.22 and 1.35 for proteins of HAdV-B3- and HAdV-C5-infected cells, respectively. 

We noticed that the iTRAQ approach quantified a larger number of proteins than the label-free method, most likely due to the prefractionation of the iTRAQ-sample. Since the sample fractionation approaches were different for the iTRAQ samples and the label-free samples, certain peptides may have not been recovered or have missed the threshold of detection in only one of the two approaches. However, to evaluate the accuracy of the iTRAQ and label-free quantitation approaches, we only compared proteins commonly quantified by both methods. 

For Sc+ against PL, the squared Pearson correlation *R* ^2^ was 0.37 for 532 proteins in HAdV-B3-infected cells, and 0.60 for 535 proteins in HAdV-C5-infected cells (Figure 3(c), S Table 1, pages 18–34). In both iTRAQ-8plex (Sc+) and label-free (PL) samples from HAdV-B3- and HAdV-C5-infected cells 20 and 9 proteins were either up- or downregulated (0.6 log_2_ ratio), respectively (S Table 2). The slope values of PL against Sc+ were 1.11 for HAdV-B3 and 1.28 for HAdV-C5-infected cells (Figure 3(c)), indicating that alterations in protein abundance due to infection were more pronounced in the label-free approach in comparison to the iTRAQ-labeling method. 

The *R* ^2^ Pearson correlation of Sc+ against PF2 was 0.54 for 394 proteins in HAdV-B3, and 0.71 for 396 proteins in HAdV-C5-infected cells (Figure 3(d), S Table 1, pages 70–82). For HAdV-B3-infected cells, 13 and 9 proteins from both infections were changed (cutoff 0.6 log_2_ ratio) in both iTRAQ-8plex (Sc+) and label-free (PF2) analyses (S Table 2). Most of the regulated commonly identified proteins were detected in the label-free approach analyzed with PF2 (S Table 2). The slope values of PF2 against Sc+ were 1.30 for HAdV-B3-, and 1.59 for HAdV-C5-infected cells (Figure 3(d)). Together, the results confirmed that the quantitation of proteins with the label-free method yielded higher expression changes than the iTRAQ-labeling quantitation for the same proteins. 

**Figure 4:**
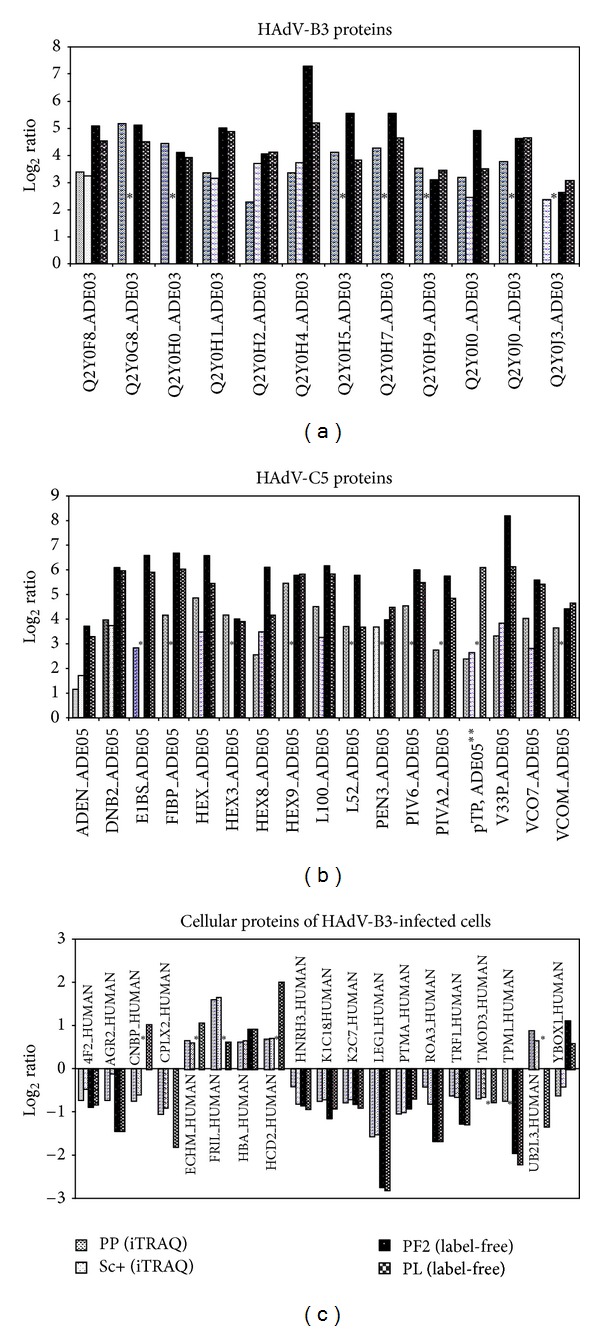
Comparison of iTRAQ-label-based versus label-free quantitations for virus-induced protein expression changes. The iTRAQ-label was based on PP and Sc+ quantitation software, while the label-free was based on PL and PF2 quantitation software. The threshold of listed proteins was set at 0.6 log_2_ ratio cutoff and included quantified viral proteins detected in HAdV-B3-infected cells (a), HAdV-C5-infected cells (b), and quantified cellular proteins detected in HAdV-B3-infected cells (c). Names of HAdV-B3 proteins, most HAdV-C5 proteins and cellular proteins correspond to accession names used in the uniprot.org database, except pTP of HAdV-C5 which was obtained from NCBI database (uniprot database does not contain this protein sequence), denoted as (∗∗). (∗) indicates that no quantitation value was obtained.

In summary, we found 12 and 17 viral proteins to be up-regulated by more than 0.6 log_2_ ratio in HAdV-B3- and HAdV-C5-infected-cells, respectively (Figures 4(a) and 4(b)). In most cases, the label-free approach revealed a higher expression change of viral proteins than the iTRAQ method. Robust expression changes were also observed for 17 cellular proteins in HAdV-B3-infected cells (Figure 4(c)), while three proteins showed inconsistent patterns in iTRAQ-label and label-free quantitations. These included cellular nucleic acid-binding protein (CNBP1), ubiquitin-conjugating enzyme E2L3 (UbcH7), and nuclease-sensitive element-binding protein 1 (YBX1). In HAdV-C5-infected cells, only two common up- or downregulated cellular proteins were observed at 24 h post infection, sequestosome-1 (SQSTM1, also known as ubiquitin binding protein p62) involved in the NF*κ*B pathway [48] and heat shock 70 kDa protein 1A/1B (HSPA1A), an ATP-dependent molecular chaperone (S Table 2). 

### 3.5. Western Blot Quantitation for Four Up- or Downregulated Cellular Proteins in HAdV-B3- or HAdV-C5-Infected Cells

**Figure 5:**
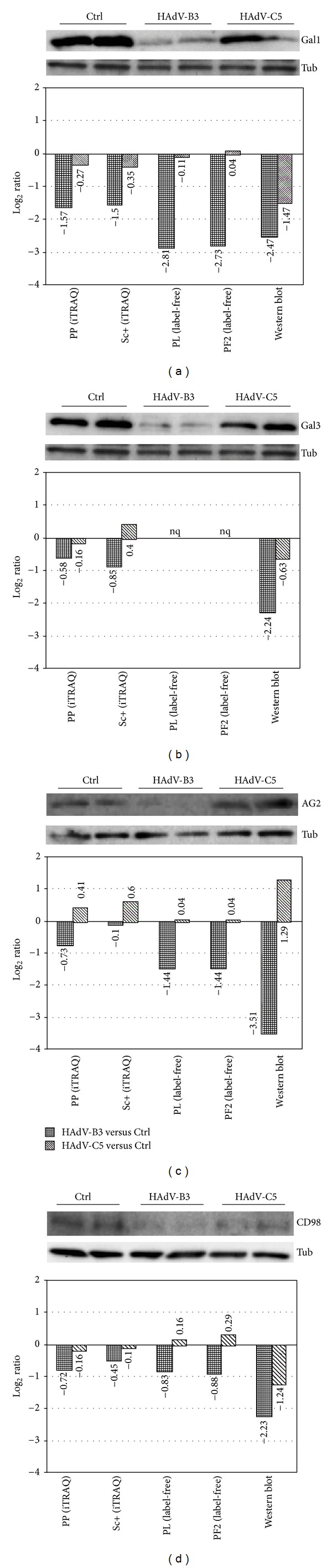
Expression analysis of five cellular proteins and comparison of different quantitation methods. Lysates from Ctrl (noninfected cells) and HAdV-B3/C5-infected A549 cells (biological duplicates) were used for Western blot analyses. Alpha-tubulin was used as a loading control for the normalization. Western blot analyses and quantitative comparisons were performed for Gal1 (LEG1_HUMAN) (a), Gal3 (LEG3_HUMAN) (b), AG2 (AGR2_HUAMN) (c), and CD98 (4F2_HUMAN) (d). nq: no quantitation value was obtained.

To validate the iTRAQ-label and label-free quantitation results, we analyzed four downregulated cellular proteins by Western immunoblotting, including galectin-1 (LGALS1, or Gal1), galectin-3 (LGAL3, or Gal3), Anterior gradient protein 2 homolog (AG2), and CD98 (SLC3A2), an activator of dibasic and neutral amino acid transporter in both HAdV-B3- and HAdV-C5-infected cells. Although Gal3 was quantified in the iTRAQ experiment but was not detected with the label-free approach, it was included in the analyses, since it belongs to the same class of lectins as Gal1 and binds to galactose of a variety of glycoproteins and glycolipids [49]. In HAdV-B3-infected cells, Gal1 was found to be downregulated in Western blots similar to the label-free analyses, while the downregulation was less pronounced according to the iTRAQ method (Figure 5(a)). In HAdV-C5-infected cells, Gal1 remained roughly constant in both iTRAQ-label and label-free quantitations but was reduced by 1.47 log_2_ ratio in Western blots. Western blots revealed a stronger downregulation of Gal3 compared to iTRAQ-label quantitation in HAdV-B3-infected cells, while the Gal3 levels in HAdV-C5-infected cells remained largely unaffected (Figure 5(b)). 

Western blot analyses confirmed downregulation of AG2 in HAdV-B3-infected cells, even more pronounced in label-free quantitation than iTRAQ quantitations (Figure 5(c)). The fold change quantified by Western blot analysis was closer to label-free quantitation than to iTRAQ ratios. In HAdV-C5, AG2 was induced in both Western blots and label-free analyses, but not in iTRAQ. Likewise, Western blot quantitations for CD98 showed robust downregulation, stronger than in label-free quantitation and even stronger than in iTRAQ samples (Figure 5(d)). In summary, the Western blot validations performed here support the notion that the protein ratios in complex mixtures are underestimated by the iTRAQ method. 

## 4. Discussion 

Increasingly, label-free quantitative proteomic methods are considered as reliable and important tools to complement labeling methods, owing to compatibility with high-throughput and high speed as well as to improved reproducibility of prefractionation of complex peptide mixtures [50]. 

Here, we performed iTRAQ experiments using a MALDI-TOF-TOF mass spectrometer and label-free experiments using an LTQ-FT-ICR instrument. For a comparison of protein quantification approaches, application of a single type of mass spectrometer would have been favorable. However, LTQ-FT-ICR instruments are not fully compatible with iTRAQ analyses because iTRAQ-reporter ions, which have a small mass per charge ratio (m/z), tend to get lost during acquisition. Conversely, MALDI-TOF/TOF mass spectrometers are not commonly utilized for label-free protein quantification. Nonetheless, we analyzed iTRAQ-labeled samples from one experiment with a 4800 MALDI-TOF/TOF (ABI) and with an LTQ Orbitrap XL (Thermo Scientific) mass spectrometer. The results of this comparison of quite different MS platforms were highly similar with regard to iTRAQ ratios (data not shown). In principle, LTQ Orbitrap XL have a similar performance to that of LTQ-FT-ICR instruments except that low mass ions including iTRAQ reporter ions can be detected by utilizing higher energy collisional dissociation. 

Our biological replicates for HAdV-B3- and HAdV-C5-infected cells showed fairly good correlations, in both iTRAQ-label-based and label-free quantitation, irrespective of the particular quantitation software or strategy used. With the same dataset obtained from iTRAQ experiments, PP searches resulted in 1,538 quantified proteins for HAdV-B3- and 1,548 proteins for HAdV-C5-infected cells with ≥95% protein confidence (1.89% FDR), while Sc+ using the Mascot search engine showed 1,340 and 1,343 quantified proteins for HAdV-B3-and HAdV-C5-infected cells, respectively, on peptide level of an ion score ≥30 (resulting in 0% FDR). Among these, 1,263 proteins in HAdV-B3- and 1,267 proteins in HAdV-C5-infected cells were commonly quantified by Sc+ and PP. The intersection could, therefore, be used to correlate the quantitative results. In the label-free method, PL quantified 661 proteins in HAdV-B3- and 660 proteins in HAdV-C5-infected cells, while PF2 quantified 439 proteins in both HAdV-B3- and HAdV-C5-infected cells. The reduction to 439 proteins can be explained by the fact that the PF2 method requires at least two peptides per protein. All proteins, which were quantified by PF2, were also quantified by PL. In this study, the peptide complexity in the iTRAQ-label approach was reduced with a prefractionation step by using SCX-HPLC prior to LC-MS/MS, while prefractionation of peptides in the label-free method was not considered prior to LC-MS/MS. Therefore, the number of proteins quantified by the label-free method was smaller than that in the fractionated iTRAQ samples. 

Changes of differentially expressed proteins were generally similar for both iTRAQ and label-free methods, either up- or downregulated. This indicates that ratiometric analyses by both iTRAQ and label-free quantitation are reliable. However, the fold changes of a large set of viral and cellular proteins tend to be larger in the label-free method. This ratio compression issue has been noticed before in studies using less complex samples than used here [19, 22, 51–53]. In our case, we have validated the results for four proteins by Western blotting (Gal1, Gal3, AG2, and CD98) and confirmed that the ratios determined by the label-free method were closer to the values from the Western blot analyses than the iTRAQ-ratios. 

### 4.1. Advantages and Drawbacks of Quantitative Proteomics Using iTRAQ and Label-Free Methods

The findings that the label-free method provided a high dynamic range and was closer to the data from Western blotting than the iTRAQ-label approach are important for studies that aim to provide high accuracies of protein ratios for different samples. The observation of underestimation of protein ratios by iTRAQ is in agreement with previous observations [19, 22, 53]. The iTRAQ quantitation method could cause the underestimation of the actual fold change by a number of reasons. (i) Precursor ions with similar masses but from different peptides could be selected for the acquisition of CID spectra. This would shift the ratio towards one and result in an underestimation of the quantitation of the peptide of interest identified in the CID spectrum. The label-free method does not have this drawback if the LCMS signals are properly aligned. (ii) Identification and quantitation are based on MS/MS data while other methods rely on both full MS and fragmentation of precursor ions. (iii) iTRAQ reagents can pose problems for certain types of mass spectrometers due to the low mass cutoff and impurities [21] which seems to be a key factor leading to underestimation of iTRAQ quantitation. (iv) In addition, incompletely labeled peptides also contribute to quantitation [20]. Despite recent improvements of iTRAQ reporter ion intensity in iTRAQ-8plex compared to iTRAQ-4plex and algorithms in PP software to filter out background signals, quantitation based on iTRAQ ratios remains problematic. 

Both iTRAQ-8plex and label-free quantitation show benefits and disadvantages. An advantage of the iTRAQ-8plex over the label-free method is the ability to analyze up to 8 samples within a single LC-MS/MS experiment, while label-free quantitation requires individual LC-MS/MS experiments and consumes more instrument time. On the other hand, the selection of samples to be compared has to be determined upfront, and the extent of multiplexing is limited.

### 4.2. Possible Biological Significance of the Results

Both the iTRAQ-based and label-free methods allowed us to identify a large number of differentially regulated proteins in HAdV-B3- and HAdV-C5-infected human lung carcinoma cells. For instance, ferritin light chain (FTL, P02792) was up-regulated 1.6- to 1.65 log_2_-fold in HAdV-B3 and 2.13- to 2.45 log_2_-fold in HAdV-C5 with PP or Sc+, respectively (S Table 2). FTL plays an important role in iron homeostasis and maintenance of iron ions in cells [54]. Another example is ITGB5 also known as *β*_5_ integrin (P18084), which was downregulated by 0.6- to 0.75 log_2_-fold in both HAdV-B3- and HAdV-C5-infected cells. ITGB5 belongs to the heterodimeric integrin protein family, and *α*_*v*_ *β*_5_ integrin functions as a receptor for fibronectin [55] and vitronectin [56]. Both *α*_*v*_ *β*_3_ and *α*_*v*_ *β*_5_ integrins together with CAR are coreceptors for HAdV-B3 and HAdV-C5 infections [40, 57–63]. In polarized human epithelial cells, *α*_*v*_ *β*_3_ is recruited from the basolateral to the apical side upon cytokine stimulation and facilitates apical infection with HAdV-C5 or HAdV-C2 [41]. In addition, high expression of ITGB5 is required for efficient HAdV-mediated gene transfer in the human airway cells [64]. 

Furthermore, Gal1 and Gal3 were both downregulated in HAdV-B3-infected cells and to a much lesser extent in HAdV-C5-infected cells. Gal1 and Gal3 are lectins which are involved in intracellular and extracellular signaling [49, 65]. Gal1 interacts with cytoplasmic and nuclear proteins to trigger multiple signaling pathways [66], and it is translocated by nonconventional mechanisms outside the cell where it binds cell surface glycoproteins and extracellular matrix components. 

In addition, Gal1 is involved in infection with Nipah virus [67] and human immunodeficiency virus (HIV) [68]. In the case of Nipah virus, Gal1 inhibits virus attachment and host cell fusion by binding N-linked oligosaccharides from the virion envelope or capsid glycoproteins and promotes their cross-linking and oligomerization. It promotes HIV infection by stabilizing virus attachment to host cells. The role for Gal1 and Gal3 in HAdV infections remains unknown. Thus, our finding that downregulation of Gal1 and Gal3 occurs in HAdV-infected cells 24 h post infection suggests a function of these proteins at mid or late stages of infection. 

Other proteins such as AG2, CD98, and immune modifier peptide thymosin alpha 1 (P06454, S Table2) were found to be downregulated in HAdV-B3-infected cells by both the iTRAQ-label and label-free approaches. AG2 expression levels were found to inversely correlate with p53 response in the preneoplastic tissue Barrett's oesophagus [69]. CD98 interacts with *β*1 integrins resulting in an increase in its affinity for integrin ligands [70]. Thymosin alpha 1 has been clinically tested in combination with other drugs to confer resistance to certain infectious agents such as hepatitis B virus [71]. The regulation of thymosin alpha 1 upon HAdV infections may be interesting for improvement of gene therapy efforts. 

We also observed a number of proteins with different regulation patterns between HAdV-B3- and HAdV-C5-infected cells. These differences could be due to the infectious pathways used by these viruses. For instance, heterogeneous nuclear ribonucleoprotein H3 (HNRNPH3, P31942), which is involved in early heat shock-induced splicing arrest [72], was downregulated in HAdV-B3-(−0.85 log_2_ ratio) and up-regulated in HAdV-C5-infected cells (0.62 log_2_ ratio). Similarly thioredoxin domain-containing protein 17 (TXNDC17, Q9BRA2), which modulates tissue necrosis factor- (TNF-) alpha signaling and NF-kappa-B activation [73], was downregulated in HAdV-B3-(−1.25 log_2_ ratio) and up-regulated in HAdV-C5-infected cells (0.7 log_2_ ratio). It has been shown previously that HAdV-2C E3-19 K protein activated the transcription factor NF-kappa-B [74]. Thus, the deregulation of TXNDC17 may be the result of an HAdV serotype-specific antiviral response. 

Overall, our findings provide a large set of proteins differentially regulated in the course of HAdV infections. This provides a basis for follow-up studies on the mechanisms by which proteins are involved in interactions of HAdV with host epithelial cells. 

## 5. Conclusions

In this study, we addressed an ongoing controversy about the most suitable methods for quantitative proteomics. We measured the relative abundance of a large number of proteins in HAdV-infected and noninfected human epithelial A549 cells using two distinct approaches, iTRAQ-based quantitation and a label-free method. In addition, we employed two independent quantitation software for each quantitation approach to reduce the bias of quantitation. The different software for the same dataset resulted in the comparable fold changes or regulation patterns. Both methods reliably determined the trends of protein up- or downregulation in virus-infected cells with overall technical replica correlation coefficients *R* ^2^ from 0.48 to 0.86. We found that the label-free method had a higher dynamic range and seemed to be more accurate than iTRAQ, which tended to underestimate the actual abundance changes of proteins. This finding was confirmed by Western blotting for four proteins downregulated particularly strongly in HAdV-B3-infected cells, Gal1, Gal3, AG2, and CD98. However, the iTRAQ approach allowed identification and quantification of a much larger number of proteins. The results suggest that the label-free method can be used as a rather accurate measure of relative protein abundance in complex mixtures of proteins. These analyses provide a basis for deeper studies of cellular and viral factors controlling virus-host interactions. 

## Supplementary Material

Supplementary Figure 1: Comparison of PF2 and PF3 for label-free quantitations. Samples were obtained from HAdV-B3-infected cells (red), and HAdV-C5-infected cells (black).
Supplementary Table 1: Pairwise comparisons for commonly quantified proteins given by log2 ratios. The ratios for the same proteins in HAdV-B3/C5-infected cells vs Ctrl were compared by different quantitation software including PL vs PP (581 proteins), PL vs Sc+ (540 proteins), PF2 vs PL (455 proteins), PF2 vs PF3 (363 proteins), PF2 vs PP (442 proteins), and PF2 vs Sc+ (1,673 proteins). (∗) indicates the value derived from the quantitation software which is used for comparison. For example, PL vs PP∗, (∗) indicates the values of quantitation obtained from the PP quantitation software.
Supplementary Table 2: List of significantly up- or down-regulated viral and cellular proteins quantified by iTRAQ and by label-free methods. Proteins with significant (Sig) fold changes higher than 0.6 log2 ratio are shown in bold. Viral proteins encoded by HAdV-B3 and HAdV-C5 appear in red and blue, respectively; cellular proteins appear in black.

## Figures and Tables

**Table 1:** Number of proteins quantified in HAdV-B3 and -C5-infected cells using different software packages.

Software package	Number of quantified proteins HAdV-B3-infected cells	Number of quantified proteins HAdV-C5-infected cells
ProgenesisF-T3PQ (label-free)	347	347
ProgenesisF-T2PQ (label-free)	438	438
Progenesis LC-MS (label-free)	660	661
ProteinPilot (iTRAQ data)	1,548	1,538
ScaffoldQ+ (iTRAQ data)	1,343	1,340

**Table 2:** Number of proteins commonly quantified with pairs of software packages.

Software packages	Number of quantified proteins HAdV-B3-infected cells	Number of quantified proteins HAdV-C5-infected cells
ProgenesisF-T2PQ versus Progenesis LC-MS	438	438
ProgenesisF-T2PQ versus ProgenesisF-T3PQ	347	347
ProteinPilot versus ProgenesisF-T2PQ	406	410
ProteinPilot versus Progenesis LC-MS	569	564
ScaffoldQ+ versus ProgenesisF-T2PQ	396	394
ScaffoldQ+ versus Progenesis LC-MS	535	532
ScaffoldQ+ versus ProteinPilot	1,267	1,263

## Abbreviations

ACN: Acetonitrile
CID: Collision-induced dissociation
DIGE: Differential gel electrophoresis
HAdV-B3: Human adenovirus type 3
HAdV-C5: Human adenovirus type 5
HPLC: High-performance liquid chromatography
ICAT: Isotope-coded affinity tag
iTRAQ: Isobaric tag for relative and absolute quantitation
LC: Liquid chromatography
LC-MS: Liquid chromatography mass spectrometry
MALDI-TOF/TOF: Matrix-assisted laser desorption/ionization time of flight
MMTS: Methyl-methanethiosulfonate
MOI: Multiplicity of infection
MS: Mass spectrometry
PAGE: Polyacrylamide gel electrophoresis
PBS: Phosphate-buffered saline
PF2: ProgenesisF-T2PQ
PF3: ProgenesisF-T3PQ
p.i.: Postinfection
PL: Progenesis LC-MS
PP: ProteinPilot
Ppm: Parts per million
RP: Reverse phase
Sc+: ScaffoldQ+
SCX: Strong cation exchange chromatography
SDS: Sodium dodecyl sulfate
SILAC: Stable isotope labeled amino acids
T2PQ: Top 2 protein quantitation
T3PQ: Top 3 protein quantitation
TandTRAQ: i-Tracker, an open-source iTRAQ quantitation
TCA: Trichloroacetic acid
TCEP: Tris[2-carboxyethyl] phosphine
FDR: False discovery rate
TFA: Trifluoroacetic acid
TNPQ: Top N protein quantification.
