# Supplementary figures and images for: iTRAQ-Based and Label-Free Proteomics Approaches for Studies of Human Adenovirus Infections
Source: Int J Proteomics. 2013 Mar 11;2013:581862. doi: 10.1155/2013/581862 (PMC3608280; doi:10.1155/2013/581862)

PF3

$\log_2(\text{HAdV-B3/Ctrl})$   
R-squared: 0.97  
Slope: 0.95

$\log_2(\text{HAdV-C5/Ctrl})$   
R-squared: 0.99  
Slope: 0.97

label-free

-5

0

5

PF2

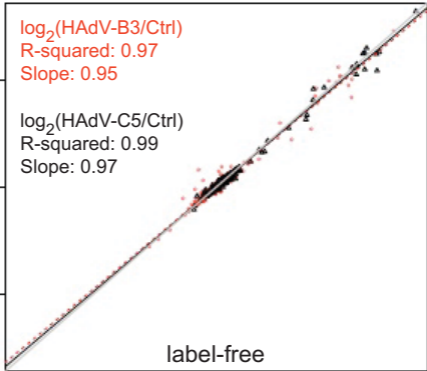

Supplement: Supplementary file 1 — Supplementary Figure 1: Comparison of PF2 and PF3 for label-free quantitations. Samples were obtained from HAdV-B3-infected cells (red), and HAdV-C5-infected cells (black). Supplementary Table 1: Pairwise comparisons for commonly quantified proteins given by log2 ratios. The ratios for the same proteins in HAdV-B3/C5-infected cells vs Ctrl were compared by different quantitation software including PL vs PP (581 proteins), PL vs Sc+ (540 proteins), PF2 vs PL (455 proteins), PF2 vs PF3 (363 proteins), PF2 vs PP (442 proteins), and PF2 vs Sc+ (1,673 proteins). (∗) indicates the value derived from the quantitation software which is used for comparison. For example, PL vs PP∗, (∗) indicates the values of quantitation obtained from the PP quantitation software. Supplementary Table 2: List of significantly up- or down-regulated viral and cellular proteins quantified by iTRAQ and by label-free methods. Proteins with significant (Sig) fold changes higher than 0.6 log2 ratio are shown in bold. Viral proteins encoded by HAdV-B3 and HAdV-C5 appear in red and blue, respectively; cellular proteins appear in black. [file 581862.f1.pdf]
